# The safety attitudes questionnaire – ambulatory version: psychometric properties of the Slovenian version for the out-of-hours primary care setting

**DOI:** 10.1186/s12913-016-1972-7

**Published:** 2017-01-13

**Authors:** Zalika Klemenc-Ketis, Matjaz Maletic, Vesna Stropnik, Ellen Tveter Deilkås, Dag Hofoss, Gunnar Tschudi Bondevik

**Affiliations:** 1 Department of Family Medicine, Medical Faculty, University of Maribor, Taborska 8, 2000 Maribor, Slovenia; 2 Department of Family Medicine, Medical Faculty, University of Ljubljana, Poljanski nasip 58, 1000 Ljubljana, Slovenia; 3 Community Health Centre Ljubljana, Metelkova 9, 1000 Ljubljana, Slovenia; 4 Faculty of Organisational Sciences, University of Maribor, Kidriceva cesta 55a, 4000 Kranj, Slovenia; 5 Health Centre Ravne na Koroskem, Ob Suhi 11, 2390 Ravne na Koroskem, Slovenia; 6 Health Services Research Unit, Akershus University Hospital, Lørenskog, Norway; 7 Institute of Health and Society, University of Oslo, Oslo, Norway; 8 Department of Global Public Health and Primary Care, University of Bergen, Bergen, Norway; 9 National Centre for Emergency Primary Health Care, Uni Research Health, Bergen, Norway

**Keywords:** Patient safety, Out-of-hours care, Quality assurance, Health care

## Abstract

**Background:**

Several tools have been developed to measure safety attitudes of health care providers, out of which the Safety Attitudes Questionnaire (SAQ) is regarded as one of the most appropriate ones. In 2007, it was adapted to outpatient (primary health care) settings and in 2014 it was tested in out-of-hours health care settings in Norway. The purpose of this study was to translate the English version of the SAQ-Ambulatory Version (SAQ-AV) to Slovenian language; to test its reliability; and to explore its factor structure.

**Methods:**

This was a cross-sectional study that took place in Slovenian out-of-hours primary care clinics in March-May 2015 as a part of an international study entitled Patient Safety Culture in European Out-of-hours services. The questionnaire consisted of the Slovenian version of the SAQ-AV. The link to the questionnaire was emailed to health care workers in the out-of-hours clinics. A total of 438 participants were invited. We performed exploratory factor analysis.

**Results:**

Out of 438 invited participants, 250 answered the questionnaire (response rate 57.1%). Exploratory factor analysis put forward five factors: 1) Perceptions of management, 2) Job satisfaction, 3) Safety climate, 4) Teamwork climate, and 5) Communication. Cronbach’s alpha of the whole SAQ-AV was 0.922. Cronbach’s alpha of the five factors ranged from 0.587 to 0.791. Mean total score of the SAQ-AV was 56.6 ± 16.0 points. The factor with the highest average score was Teamwork climate and the factor with the lowest average was Job satisfaction.

**Conclusions:**

Based on the results in our study, we cannot state that the SAQ-AV is a reliable tool for measuring safety culture in the Slovenian out-of-hours care setting. Our study also showed that there might be other safety culture factors in out-of-hours care not recognised before. We therefore recommend larger studies aiming to identify an alternative factor structure.

## Background

Patient safety is an important aspect of quality assurance and improvement in health care. In recent years, a concept of patient safety culture has been developed. It describes leader and staff interaction, attitudes, routines, awareness, and practices, which impinge on the risk of patient adverse events [1]. Safety culture is regarded as a group phenomenon rather than that of individuals [2]. In organizational psychology research, safety culture is described by both qualitative and quantitative methods [3]. Quantitative surveys have concentrated on measuring staff perceptions, which are referred to as organizational climates. Organizational climates are mathematical expressions of how members in natural social units perceive that cultural norms are enacted by leadership and members in the unit. These climates are measured both according to level of mean and the degree to which staff share the perceptions, which is the organizational climate strength [4]. Organizational climates with diverging perceptions amongst staff are regarded as weak with limited power to predict staff practices [5].

Primary health care differs from hospitals in terms of organisational structure, administrative and clinical processes and the reasons for encounters [6]. Therefore, also patient safety culture dimensions could differ when compared to secondary or tertiary health care.

So far, several studies have addressed the issue of patient safety culture in primary health care [7–10]. The revealed dimensions were similar but not identical. The most comprehensive view was provided by Kirk et al. [10] who conducted a literature review of patient safety dimensions and put forward nine dimensions that patient safety culture in primary care was expressed by: overall commitment to quality, priority given to patient safety, perceptions of the causes of patient safety incidents and their identification, investigating patient safety incidents, organisational learning following a patient safety incident, communication about safety issues, personnel management and safety issues, staff education and training about safety issues, and teamworking around safety issues.

Out-of-hours care (OOHC) services are primary health care services enabling access to primary health care services during out-of-hours working hours (weeknights and weekends) [11]. There are many different models for organizing OOHC services [11] and yet no clear definition of OOHC has been developed in the literature [12].

In Slovenia, OOHC and emergency medical services (EMS) are combined and are available both at the same place and time. Professionals that work in OOHC are family physicians, emergency physicians, and emergency nurses. Sometimes, there are also laboratory technicians and radiology technicians. Usually, emergency nurses work only in OOHC settings while family physicians work in their practice and in OOHC on the basis of rotation. One or two teams are on call at the same time on 8-hours rotation. An OOHC team consists of a family- or emergency physician and two emergency nurses. These teams are located in primary health care centres and available 24/7 enabling free access of patients on their own demand. If there is an on-the-field emergency, the team leaves the OOHC surgery in order to respond to the call. This is a major difference between Slovenian and most European OOHC centres. In a majority of European countries OOHC centres are organized separately from EMS [11]. An OOHC centre in Slovenia has thus a unified leadership and can be seen as a “natural social unit”; which is a validation criteria for organizational climate measurements [2]. Patient safety in OOHC has been studied only in few European countries [9, 13–16]. These studies dealt with several aspects of patient safety, i.e. patient safety incidents [15] and triage [13].

Several tools have been developed to measure safety attitudes of health care providers [7, 17–22]. The Safety Attitudes Questionnaire (SAQ) is one of the widely used and also one of the most appropriate instruments for measuring patient safety culture [22–24]. It measures six factors: Teamwork climate, Safety climate, Job satisfaction, Perceptions of management, Working conditions and Stress recognition [25]. In 2007, the SAQ was adapted to outpatient (primary health care) settings [7]. This SAQ – Ambulatory Version (SAQ-AV) proved to be a reliable tool for comparing attitudes across different professional groups of health care providers outside hospitals [7]. In 2014, Bondevik et al. [16] tested the Norwegian version of SAQ-AV and proved it to be a reliable tool for measuring patient safety culture in OOHC in Norway.

In Slovenia, patient safety culture in OOHC has not been studied and no tools for measuring it have yet been developed or adapted. The purpose of this study was to translate the SAQ-AV to Slovenian language and to adapt it to the Slovenian OOHC settings. We had the following aims of the study: 1) to translate the English version of SAQ-AV to Slovenian language; 2) to test the reliability of the Slovenian version of SAQ-AV in OOHC settings; and 3) to determine the factor structure of the Slovenian version of SAQ-AV in OOHC settings.

## Methods

### Type of study and settings

This was a cross-sectional study that took place in Slovenian out-of-hours primary care clinics from March 16^th^ to May 1^st^ 2015. The study was a part of an international study entitled Patient Safety Culture in European Out-of-hours services (SAFE-EUR-OOH), which was led by a coordinating research group from Norway. It was a project of the European research network for out-of-hours primary health care (EurOOHnet) [26].

In Slovenia, there are 60 out-of-hours clinics. All were invited, and 37 (61.7%) agreed to participate. These OOHC clinics were of variable size; the smallest covered 35,000 inhabitants and the largest 300,000 inhabitants. On average 30 professionals work in these clinics.

### Research instrument

We used the SAQ-AV which was translated from English to Slovenian according to modified principles adapted from Beaton et al. [27]. Initially, the original English version was translated into Slovenian using a professional translation bureau. Next, an expert committee with Slovenian clinicians and researchers adapted the initial translated version to the OOHC setting in Slovenia. This adapted version of the questionnaire was translated back into English by a second independent translation bureau being blinded to the original version. Based on this back-translated version, the national expert committee made the necessary adjustments in order to clarify possible misunderstandings. The pre-final version was evaluated by six employees in different OOHC clinics. They were asked to give feedback about the comprehensibility of the Slovenian version of SAQ-AV. Minimal changes to the questionnaire regarding Slovenian wording were made according to their suggestions. These six employees were later invited to participate in the study.

The SAQ-AV consists of 62 items. Each item should be answered on a 5-point Likert scale by which the respondents indicated their level of agreement with the statement (1 = disagree strongly, 2 = disagree slightly, 3 = neutral, 4 = agree slightly, 5 = agree strongly) [7]. In the analysis, scores of negatively worded items were reversed, so that higher scores in the data set always indicate a more positive evaluation of the unit’s patient safety culture.

There were also some demographic questions (sex, age, function, working experiences, shifts, type of employment).

### Data collection

In each Slovenian OOHC clinic that agreed to participate, a person in charge for the data collection was selected. This person asked all employees (physicians, graduate nurses, nurse managers, trainees, nurses, radiology technicians, and office managers) working in the OOHC clinic to participate. Using this procedure, a total of 438 people agreed to participate. The participation was voluntary.

The national key researcher for Slovenia (ZKK) collected the e-mail addresses of all willing employees in participating OOHC clinics. On March 16^th^ 2015, the link to an electronic version of the Slovenian SAQ-AV was mailed to all participants from the coordinating research group in Norway, using the data collection program Qualtrics. An automatic reminder was sent by Qualtrics to those who had not responded after two weeks.

The Qualtrics file with SAQ-AV data from Slovenia was converted into SPSS for further analysis. The researchers were provided with anonymous data files where possible identifiers like e-mail and IP addresses had been removed by the administrative coordinator in the project. It was not possible for the researchers to link participants to their responses.

### Statistical analysis

We performed the explorative factor analysis (EFA) with the aim of data reduction and therefore simplification of a large number of intercorrelated measures of safety attitude to a few representative constructs or factors. Out of 62 items of the SAQ, 31 items were entered into the EFA. The 31 items corresponds to the measurement model of SAQ which was tested and validated in a previous study [9]. Further, the principal components analysis (PCA) with the Varimax rotation method was applied to discover the main patterns of variation among respondents in accordance with the dimensions identified by the EFA. First, mean scores were calculated from the scale’s items to generate the composite scores for the safety attitude dimensions. The newly created composite variables were subject of the PCA. Scale reliability was tested by Cronbach’s alpha. We also determined the K-M-O and Bartlett statistic. Additionally, we performed corrected item-total correlations (CITCs) in order to strengthen validity and reliability results.

A free software environment for statistical computing and graphics R was applied using the prcomp() function in the stats package with the purpose of applying PCA and visualizing the data by using the biplot.

We decided to retain the five factors which had eigenvalues greater than one.

## Results

### Demographic characteristics of the sample

Out of 438 invited participants, 250 answered the questionnaire (response rate 57.1%). The demographic characteristics of the sample are presented in Table 1.

**Table 1:** Demographic characteristics of the participants

Characteristic	N (%)
Sex	
Male	91 (36.4)
Female	110 (44.0)
Missing	49 (19.6)
Age (years)	
30 and lower	41 (16.4)
31–40	74 (29.6)
41–50	49 (19.6)
51–60	33 (13.2)
61 and higher	4 (1.6)
Missing	49 (19.6)
Usual shift	
Days	3 (1.2)
Evenings	2 (0.8)
Nights	4 (1.6)
Variable	192 (76.8)
Missing	49 (19.6)
Job status	
Full-time	191 (76.4)
Part-time	7 (2.8)
Contract	3 (1.2)
Missing	49 (19.6)
Function	
Physicians	93 (37.2)
Graduate nurses	43 (17.2)
Nurse managers	3 (1.2)
Trainees	15 (6.0)
Nurses	40 (16.0)
Radiology technicians	1 (0.4)
Office managers	7 (2.8)
Missing	48 (19.2)

### Factor structure of the SAQ

Due to incomplete SAQ-AV responses, 119 (47.6%) cases were excluded from the factor analysis. EFA put forward five factors: 1) Perceptions of management, 2) Job satisfaction, 3) Safety climate, 4) Teamwork climate, and 5) Communication. Perceptions of Management included six items, Job Satisfaction six items, Safety Climate four items, Teamwork Climate three items, and Communication three items (Table 2).

**Table 2:** Factor model and reliability

Item	Cronbach’s alpha	Factor loading	CITC
Factor 1: Perceptions of management	0.765		
Senior management of this office is doing a good job.		0.710	0.616
The management of this office supports my daily efforts.		0.611	0.569
The levels of staffing in this office are sufficient to handle the number of patients.		0.582	0.416
This office is a good place to work.		0.582	0.542
I receive appropriate feedback about my performance.		0.561	0.565
This office deals constructively with problem personnel.		0.543	0.390
Factor 2: Job satisfaction	0.791		
It is easy for personnel in this office to ask questions when there is something that they do not understand.		0.628	0.583
The culture in this office makes it easy to learn from the errors of others.		0.611	0.498
I am proud to work at this office.		0.560	0.641
I have the support I need from other personnel to care for patients.		0.526	0.395
Disagreements in this office are resolved appropriately (i.e., not who is right but what is best for the patient).		0.517	0.599
Working in this office is like being part of a large family.		0.508	0.564
Factor 3: Safety climate	0.761		
All the necessary information for diagnostic and therapeutic decisions is routinely available to me.		0.747	0.547
Medical errors are handled appropriately in this office.		0.718	0.625
I know the proper channels to direct questions regarding patient safety in this office.		0.615	0.583
During emergencies, I can predict what other personnel are going to do next.		0.549	0.502
Factor 4: Teamwork climate	0.587		
Nurse input is well received in this office.		0.681	0.499
I like my job.		0.564	0.327
Attending physicians/primary care providers in this office are doing a good job.		0.479	0.395
Factor 5: Communication	0.685		
I am frequently unable to express disagreement with staff physicians/intensivists in this office.		0.766	0.499
In this office, it is difficult to discuss errors.		0.745	0.535
In this office, it is difficult to speak up if I perceive a problem with patient care.		0.539	0.464

*CITC* corrected item-total correlations

### Reliability of the SAQ and its factors

Cronbach’s alpha of the five factors ranged from 0.587 to 0.791. Those five factors accounted for 52.7% of the variance (K-M-O statistic 0.897; Bartlett statistic 2378.201 (*p* < 0.001). The corrected item-total correlation scores of the factors ranged from 0.39 to 0.64 (Table 2).

**Fig. 1:**
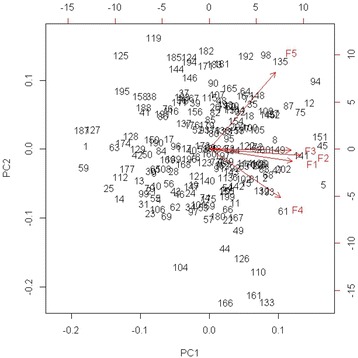
The principal component analysis (PCA) biplot

The bi-plot (the bivariate plot) in Fig. 1 shows a strong relationship between the Perceptions of management, Job satisfaction and Safety climate. It should be noted that all variables point to the same direction, which indicate that variables are positively associated. The eigenvalues indicate that two components provide a reasonable summary of the data, accounting for 69.65% of the total variance. From the PCA bi-plot (Fig. 1) it can be observed that the variance along the Comp. 2 axis is higher than along Comp. 1 axis, especially if the account outliers (e.g. 161, 166 and 133) are taken into account. For example, in this case, outliers have significantly lower values corresponding to the Communication (F5) dimension of the safety attitude construct. In contrast, the respondents who are plotted in upper part of the biplot (positive loadings on Comp. 1 and Comp. 2) expressed higher level of agreement with the Safety climate, Teamwork climate and Communication. Moreover, the density of the respondents is a bit higher around the centre of the bi-plot (at the starting point of the vectors). It appears that those respondents expressed moderate agreement with the Perceptions of management, Job satisfaction and Safety climate.

## Discussion

The Slovenian version of SAQ-AV, EFA revealed five factors, some of which were similar, but not identical to previous versions of the SAQ-AV [7, 16]. This study was a part of the international study lead by the Norwegian researchers who had adapted the SAQ-AV for Norwegian primary care settings. The Norwegian factor structure [16] provided five factors: Teamwork climate, Safety climate, Job satisfaction, Working conditions, and Perception of management. The original SAQ, developed at the University of Texas at Austin [25], described six factors: Teamwork climate, Safety climate, Working conditions, Job satisfaction, Perceptions of management and Stress recognition.

Two of the factors in our study had Cronbach’s alphas lower than the recommended 0.7 [28]. Considering cultural differences between the countries this is not surprising. Although content of four factors had similarities in the Norwegian and Slovenian factor structure, several items loaded on different factors in Slovenia compared to Norway.

This indicates that the perceptions of safety culture in Norway and Slovenia may be different. Items related to perceived problems voicing concerns regarding patient safety, emerged as an independent factor in our study. We named this factor Communication. Items included in this factor in Slovenia belong to several factors found in other studies [25, 29]: Teamwork climate, Safety climate, and Perceptions of management. Actually, Communication is a part of all these fields but it seems that it was so important to the Slovenian participants that it emerged as an individual factor.

There is some overlap between Communication and the concept of psychological safety. The term psychological safety refers to which extent team members feel comfortable seeking feedback, sharing information, asking for help, talking about errors, and experimenting [30–32]. Psychological safety is a requirement for effective communication regarding risk in patient care [31], and is complementary to a good safety culture [33]. Psychological safety has been recognised as safety relevant in other health care settings [30, 33] but not in previous analyses of SAQ-AV data nor in OOHC settings. Our results may indicate that Communication and psychological safety are perceived more important and perhaps cultural traits more at stake to participants in Slovenia compared to other countries, since they possibly emerge as individual factors without the study seeking to map them explicitly. The finding supports that Communication and Psychological safety are considered possible independent factors in future safety culture surveys.

Our study has some methodological limitations which need to be addressed. The first one is the process of factor analysis. We chose not to proceed by confirmatory factor analysis despite the fact that it is usually a key step in psychometric evaluation of survey tools. Our decision was based on small sample size which was a consequence of specific study settings.

The second one is the low value of Cronbach’s alpha in two of the factors. These are the same factors that have only three items. As the alpha value is related to the number of items in the scale [34], the low values of Cronbach’s alpha could be a consequence of the inclusion of a limited number of items. These two factor were also problematic in terms of item-to-total correlations, which were below the recommended 0.5 [28]. However, some authors advocate the cut-off value of 0.3 [35]. These factors were not removed from the model and the decision was based on content consideration and acceptable alpha values. The exclusion of items did not improve the model.

Another limitation is the limited response rate and the relatively high number of incomplete questionnaires. However, only two clinics out of 37 failed to provide at least one complete questionnaire. After the exclusion of 119 incomplete questionnaires, 131 cases were available for factor analysis of the selected 22 variables. As noted by Mundfrom et al. [36] there are no clear criteria for deciding the appropriate factor analysis sample size. Suggestions for minimum absolute sample size range from 100 to over 1000, and suggested minimum ratio of number of variables to number of factors range from 3 to 20, depending, among other things, upon the number of factors extracted and the level of communality [37]. The sample size in our study was limited, and we recommend further studies with a higher number of participants.

An EFA always produces a solution, but does not assess the risk that the EFA solution only describes the data set, and may not be generalizable. Due to these limitations, the results of our study may not be generalised to the whole population.

## Conclusions

Based on the results in our study, we cannot state that the SAQ-AV is a reliable tool for measuring safety culture in the Slovenian out-of-hours care setting. This might be due to methodological limitations. Our study also showed that there might be other safety culture factors in OOHC not recognised before, such as psychological safety. We therefore recommend larger studies aiming to identify an alternative factor structure and other concepts of safety culture in OOHC.

## Abbreviations

CFA: Confirmatory factor analysis
CITs: Corrected item-total correlations
EFA: Explorative factor analysis
EMS: Emergency medical services
EurOOHnet: European research network for out-of-hours primary health care
OOHC: Out-of-hours Health Care
SAQ: Safety attitudes questionnaire
SAQ-AV: Safety attitudes questionnaire-ambulatory version
